# Novel composite materials of modified roasted date pits using ferrocyanides for the recovery of lithium ions from seawater reverse osmosis brine

**DOI:** 10.1038/s41598-021-98438-2

**Published:** 2021-09-23

**Authors:** Rana S. Al-Absi, Mohammed H. Abu-Dieyeh, Radhouane Ben-Hamadou, Mustafa S. Nasser, Mohammad A. Al-Ghouti

**Affiliations:** 1 Department of Biological and Environmental Sciences, College of Arts and Sciences, Qatar University, P.O. Box: 2713, Doha, State of Qatar Qatar; 2 Gas Processing Center, College of Engineering, Qatar University, Doha, State of Qatar Qatar

**Keywords:** Environmental sciences, Chemistry

## Abstract

In this paper, novel composite materials from modified roasted date pits using ferrocyanides were developed and investigated for the recovery of lithium ions (Li^+^) from seawater reverse osmosis (RO) brine. Two composite materials were prepared from roasted date pits (RDP) as supporting material, namely potassium copper hexacyanoferrate-date pits composite (RDP-FC-Cu), and potassium nickel hexacyanoferrate-date pits composite (RDP-FC-Ni). The physiochemical characterization of the RO brine revealed that it contained a variety of metals and salts such as strontium, zinc, lithium, and sodium chlorides. RDP-FC-Cu and RDP-FC-Ni exhibited enhanced chemical and physical characteristics than RDP. The optimum pH, which attained the highest adsorption removal (%) for all adsorbents, was at pH 6. In addition, the highest adsorption capacities for the adsorbents were observed at the initial lithium concentration of 100 mg/L. The BET surface area analysis confirmed the increase in the total surface area of the prepared composites from 2.518 m^2^/g for RDP to 4.758 m^2^/g for RDP-FC-Cu and 5.262 m^2^/g for RDP-FC-Ni. A strong sharp infrared peak appeared for the RDP-FC-Cu and RDP-FC-Ni at 2078 cm^−1^. This peak corresponds to the C≡N bond, which indicates the presence of potassium hexacyanoferrate, K_4_[Fe(CN)_6_]. The adsorption removal of lithium at a variety of pH ranges was the highest for RDP-FC-Cu followed by RDP-FC-Ni and RDP. The continuous increase in the adsorption capacity for lithium with increasing initial lithium concentrations was also observed. This could be mainly attributed to enhance and increased lithium mass transfer onto the available adsorption active sites on the adsorbents’ surface. The differences in the adsorption in terms of percent adsorption removal were clear and significant between the three adsorbents (*P* value < 0.05). All adsorbents in the study showed a high lithium desorption percentage as high as 99%. Both composites achieved full recoveries of lithium from the RO brine sample despite the presence of various other competing ions.

## Introduction

There has been a continuous increase in populations, development, urbanization, and industrialization in the world^1^. These human-derived factors impact the availability of fresh water resources^2^. A well-established method for providing fresh water is seawater desalination, which is a process that produces potable water from seawater. Many countries, like Qatar, invest substantial amounts annually of their technological and financial resources in desalination research, training, and implementation to overcome their water scarcity problems^3–5^. Zheng and coworkers reported that during the last two decades, a major worldwide trend in employing desalination technologies to overcome the water availability issues has been noticed^6^. The number of countries relying on desalination to a great extent remained to increase continuously after that. For example, it was estimated that in the year 2011, more than 30 million m^3^/d of desalinated potable water was produced globally. In addition, the economic trends of the world’s spending on desalination technologies revealed that by the year 2015 the desalination industry would reach 30 billion US dollars^5,7^. In the year 2015, it was reported that around 18,426 desalination plants existed around the world, which provided more than 150 countries with more than 86.8 million m^3^ of fresh water. Interestingly, the North African and Middle Eastern countries compromised around 45% of the desalination freshwater capacity provided then^8^. Specifically, seawater desalination accounts for 99% of Qatar’s accessible water, whereas groundwater meets the remaining 1%. The water per capita consumption rates in Qatar are among the highest in the world at about 500 Liters per day^4^. These consumption rates are met by increasing the number of desalination plants and capacities throughout the years. For example, in 2014, the production of desalinated potable water in Qatar was around 493 million m^3^. In 2017, the amount of desalinated water in Qatar was estimated to be 540 million m^3^^3,9^. In the year 2017, the number of countries that applied desalination technologies increased to reach 231 countries. One of the most important driving forces to this trend is the fact that water demand is also increasing^6^.

Like any industrial process, desalination technologies not only vary in their working principles but also in the produced outcomes. To be more specific, all desalination plants are based on the principle of de-salting or removing salts from seawater or brackish waters to produce fresh waters as a mainstream and brine solution as a secondary stream^10^. However, their process requirements, as well as environmental impacts vary from being extremely inefficient to being efficient^11^. Reverse osmosis (RO) is the leading membrane-based seawater desalination technology in the GCC. This is because of the ease of maintenance, simpler design, low cost, and environmental impacts as well as flexibility compared to other popular thermal desalination technologies^11^. The current conventional desalination plants employ various types of pre-treatment chemicals such as coagulants and flocculants, which could significantly harm the environment through leaks, or improper disposal of waste streams like brine. This stream is a concentrated by-product salt stream from desalination technologies, which contains various substances such as pre-treatment chemicals, salts, metals, and others^12^. A typical brine stream would be characterized by high temperatures, altered pH values, and high salinity. Consequently, the world is in need of water sustainability as well as environmentally friendly approaches in meeting the freshwater needs of the ever-growing populations^13^. This could be achieved by employing the technique of adsorption to remove, remediate and recover harmful and valuable substances from desalination brine streams. The adsorption technique is based on the use of a solid material referred to as an adsorbent that receives the target mineral known as adsorbate on its surface^14^. Many factors like adsorbent characteristics, pollutant concentration, and others can significantly affect the level of adsorption capacity^15^.

There are a variety of well-established adsorbents for the remediation of pollutants and recovery of valuable metals. Examples of these adsorbents include chitosan, alumina, and activated carbon. Many of these adsorbents must undergo a variety of modification processes to show high adsorptive and recovery capacities. These modifications are usually costly and involve the usage of harmful chemicals^16^. Scientists are seeking natural-based, cost-friendly and effective adsorbents that do not pose risks to the environment. Qatar is one of the leading countries in the production of dates and this sector produces massive amounts of date pits as waste annually. Date pits could demonstrate great adsorptive potentials towards valuable metals from brines due to their carbonaceous compositions and porous structures^15^. As mentioned previously, Qatar relies heavily on desalination to meet its national fresh water needs. Therefore, date pits could be sustainably used to remediate the reverse osmosis brine streams produced from desalination plants^17^. This would facilitate the effective, cost-efficient, and environmentally friendly remediation of two of the major waste products namely, metals from brines and date pits. Date pits could be used as support materials for more effective adsorbents like metal hexacyanoferrates. These are coordination polymers that contain coordinated bridges of C≡N and a transition metal ion like Cu^2+^, Fe^3+^, Co^2+^, and Ni^2+^^18^. Metal hexacyanoferrates have a unique cubic lattice structure that would facilitate the adsorption of valuable metals by an ion-exchange mechanism between the transition metal and the target metal^19^. These materials are commonly applied in metal extraction, recovery, and adsorption due to their insolubility, high selectivity, and adsorptive capacities. Almost all the synthesis methods of metal hexacyanoferrates are cost-effective and simple, with the most employed method is initiating a precipitation reaction between the precursors of hexacyanoferrate and the transition metals^20^.

However, according to the current study, combining the adsorptive capabilities of date pits with metal hexacyanoferrates could further provide a more efficient and cost-friendly approach in decreasing date pit waste, as well as recovering valuable metals such as lithium from RO brine. Nowadays, lithium is used in a variety of fields like lithium-ion batteries, greases, polymers, ceramics, and metal additives^21^. Due to the high demands on lithium-containing products, their extraction from hard rock ores or brine is becoming more expensive. However, the extraction of lithium from brines remains cheaper and simpler than hard rock^22^. Therefore, extracting lithium ions from reverse osmosis desalination brine would bring forward many advantages and flexibilities to Qatar and other countries to recover valuable metals from RO brine.

In this paper, novel composite materials from modified roasted date pits using ferrocyanides were developed and investigated for the recovery of the valuable metal ion, Li^+^, from seawater reverse osmosis (RO) brine. Roasted date pits (RDP) were utilized as support materials for the preparation of two composite adsorbents, namely potassium copper hexacyanoferrate-date pits (RDP-FC-Cu), and potassium nickel hexacyanoferrate-date pits (RDP-FC-Ni). A summary of the physiochemical characteristics of the RO brine was also discussed.

## Materials and methods

### Physical and chemical characterization of the collected brine

The brine samples were collected from a reverse osmosis desalination plant in Qatar. To ensure homogenous and precise measurements, replicate samples were collected from the reverse osmosis desalination plant at different periods. Moreover, the collected brine samples were mixed, and the representative sample was stored in plastic bottles in a dry, clean and dark area to prevent any contaminations or reactions with the surroundings. The brine sample’s physical parameters like pH, conductivity, salinity, and total dissolved solids (TDS) were analyzed. Moreover, inductively coupled plasma optical emission spectroscopy (ICP-OES, PerkinElmer Optima 5300 DV) and ion chromatography (IC) were used to determine the metals in the brine sample.

### Supporting material for the modification purposes

Date pits were collected from Qatari local markets and were used as supporting material for ferrocyanide modification. All the experiments involving plants adhered to relevant ethical guidelines on plant usage. The date pits were washed with distilled water to remove any impurities and dried at 100 °C for 24 h. The date pits were then roasted on a hot plate with continuous mixing at 100 °C for 6 min or till they obtained a golden-brown color. The roasting process converts most of the raw material into a rich carbonaceous material that supports high adsorption capabilities^16^. After that, the roasted date pits (RDP) were ground and sieved into different particle sizes (100–250 µm, 250–500 µm, and 500–750 µm). The date pits were then stored in glass bottles for the batch adsorption experiments.

### Ferrocyanide-date pits modification

Cu–K or Ni–K ferrocyanide was used in this study as they have high selectivity towards lithium due to ion exchange, metal complexation, and electrostatic attractions^23^. The roasted date pits-composite ferrocyanide adsorbents with Cu–K or Ni–K were then prepared^23^. First, 1 molar of K_4_[Fe(CN)_6_] (Riedel–de Haen, Germany), CuSO_4_ (Scharlau, Scharlab S.L, Spain), and NiCl_2_ (Riedel–de Haen, Germany) were prepared. Then 1:1 molar ratio of K_4_[Fe(CN)_6_] and (CuSO_4_ or NiCl_2_) were thoroughly mixed. A NaOH (RESEARCH-LAB FINE INDUSTRIES, Mumbai 400002 (India)) solution of RDP was added into the resulting suspension in sequence with strong stirring. The NaOH solution prepared was of 0.1 molar and 100 mL of the solution was added to 50 g of RDP. The NaOH-RDP solution was added into the resulting suspensions of copper ferrocyanide and nickel ferrocyanide in sequence with strong stirring. The suspension was centrifuged at 5500 rpm for 20 min, removing the supernatant, and centrifuge again for 20 min then removes the supernatant then drying the precipitate at 100 °C for 3 h. using an oven. The samples were denoted as RDP-FC-Cu and RDP-FC-Ni, respectively.

### Characterization of the adsorbents

The roasted date pits and the prepared composites were physiochemically characterized using various analytical techniques to investigate their adsorptive capabilities and characteristics. The physical characterization tests involved scanning electron microscopy to evaluate the surface morphology of the materials before and after adsorption using the NovaTM Nano SEM 50 Series, from FEI Company. A particle size distribution (FRITSCH’S ANALYSETTE 22 NanoTec) was conducted for the adsorbents to analyze their size range and their role in the adsorption process. The surface area and pore size distribution were carried out using Brunauer–Emmett–Teller (BET) from Quantachrome Corporation, Nova 3000. The chemical characterizations included the elemental analyzer to determine the chemical composition of the materials in terms of carbon and hydrogen, X-ray diffraction (XRD) (PANalytical Empyrean/Netherland) analysis as well as the Fourier-transform infrared spectroscopy (FTIR) analysis using FTIR Perkin Elmer Model 2000. The FTIR analysis was carried out to interpret the functional groups, which occurred in the roasted and the modified form as well as changes due to adsorption. The FTIR measurements were performed over 4000–400 cm^−1^. Furthermore, the thermal stability of the adsorbents was determined using thermogravimetric analysis (TGA) (PerkinElmer, Pyris 6 TGA) to investigate the durability of the adsorbents in industrial applications and large-scale adsorption processes. The TGA was done by applying argon gas and heating the samples from 25 to 800 ℃.

### Adsorption studies

The standard lithium solution (100 mg/L Li^+^) from LiCl was prepared by dissolving 0.6115 g of lithium chloride (LiCl) (Research-Lab Fine Industries, Mumbai 400002 (India)) and diluted it to one liter with distilled water. The standard lithium solution contains only lithium ions. The final concentration of the metal was in the range of 5–100 mg/L for each batch experiment. A mass of 0.05 g of the adsorbent [RDP, RDP-FC-Cu, or RDP-FC-Ni)] was placed into 50 mL of the lithium solution of known initial concentration using a polycarbonate Erlenmeyer flask. The mixture was agitated at 160 rpm in an incubator shaker for 24 h at room temperature (25 ℃) to reach equilibrium. The adsorbent was then be filtered out using a filter paper (0.2 µm). The residual lithium concentration in the solution was analyzed using ICP-OES analytical technique. Another key parameter than the effect of lithium concentration was also performed. The effect of initial solution pH on the adsorption of lithium was determined. The initial solution pH ranges were 2, 4, 6, 8, and 10. The pH of the solutions was adjusted using minute amounts of 0.5 M HCl and 0.5 M NaOH solutions. Furthermore, the zeta potential test was performed at pH of 2, 6, and 8. The zeta potential was tested using the Malvern ZETASIZER Nano series. The desorption of lithium from the three adsorbents using 0.5 M and 1 M HCl solutions was studied. The competitive adsorption of lithium from the collected RO brine was also considered.

The adsorption capacity and adsorption removal were obtained using Eqs. (1) and (2). The quantity of adsorbate that an adsorbent can carry per unit mass is known as the adsorption capacity (q_e_). The adsorption capacity is usually calculated and determined through a mathematical formula shown in Eq. (1)^24^.

1 $$ {\text{q}}_{{\text{e}}} = \frac{{\left( {C_{i} - C_{e} } \right)V}}{m} $$

where C_i_ and C_e_ are the initial and equilibrium lithium concentration (mg/L), respectively. V is the volume (L) of the solution and m is the mass (g) of the adsorbent used.

Moreover, the percentage removal of lithium was calculated as the adsorption removal, which gives insights regarding the capability of an adsorbent in adsorbing a given pollutant in terms of a whole percentage. The percentage lithium removal was calculated and determined by Eq. (2)^25^:

2 $$ {\text{\% }}\;{\text{Adsorption}}\;{\text{removal }} = { }\frac{{C_{o} - C_{e} }}{{C_{o} }} \times 100 $$

where C_o_ is the lithium concentration in mg/L before adsorption, C_e_ is the lithium concentration after adsorption is completed in mg/L.

## Results and discussion

### Physiochemical characterization of the collected reverse osmosis brine

The IC results revealed that the order of the major cations present in the brine solution in terms of decreasing concentration is as follows: calcium, sodium, magnesium, and potassium. On the other hand, the brine sample contained three major anions in the decreasing concentration order as chloride, sulfates, and bromide. The ICP-OES results showed the presence of various trace, rare, and industrially valuable elements. These elements are (in a decreasing concentration order) strontium, zinc, vanadium, lithium, iron, lead, lanthanide, cesium, and barium. Moreover, the brine was observed to be slightly alkaline as indicated by the pH, which was around 8. The salinity, electrical conductivity, and total dissolved solids (TDS) of the brine at the study were found to be 61.4 ppm, 99.5 mS/cm, and 113.17 mg/L, respectively.

### Effect of solution pH and zeta potential on the adsorption of lithium onto roasted date pits and composites

**Figure 1:**
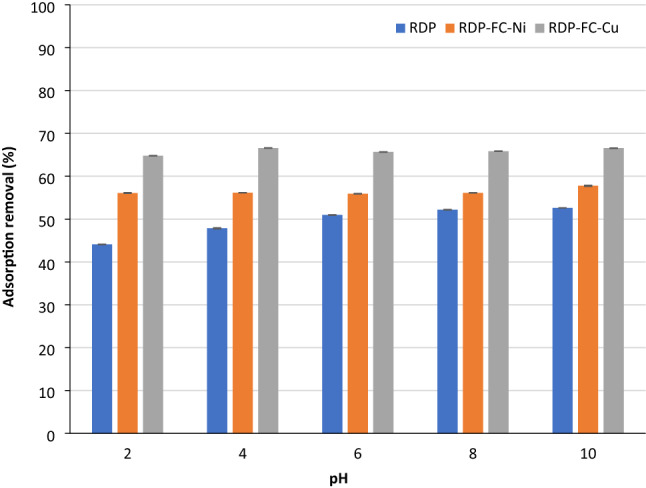
The effect of pH on the adsorption of lithium onto RDP, RDP-FC-Cu, and RDP-FC-Ni at different pH values. The experimental conditions were as follows: temperature of 25 ℃, 50 mL volume, 100 mg/L lithium-ion concentration, shaking time of 24 h. at 160 rpm, and 50 g of the adsorbent. Error bars are shown in the figure.

Figure 1 shows the adsorption removal (%) of RDP, RDP-FC-Cu, and RDP-FC-Ni towards Li^+^ at a variety of pH values. The examined pH values were 2, 4, 6, 8, and 10 while the concentration of the lithium ions was fixed to 100 mg/L. In general, it is clear from Fig. 1 that the adsorption removal for lithium follows a similar trend for all adsorbents at all pH values. The adsorption capacity of lithium at all pH values is lowest for the RDP and highest for RDP-FC-Cu (*P* value < 0.05). As mentioned previously in the FTIR results, the modification of RDP into RDP-FC-Cu and RDP-FC-Ni gave rise to characteristic and unique functional groups to the RDP. For example, the presence of C≡N, Fe–C, C-O, and C-N bonds on the composites could have enhanced their adsorptive capabilities, functionality, structure, and characteristics towards lithium.

Lithium adsorption onto the RDP (Fig. 1) shows a continuously increasing trend as the pH increases from 2 to 10 (*P* value < 0.05). The lowest adsorption removal (44.1%) for lithium on RDP was observed at pH 2. Oppositely, the highest adsorption removal (52.6%) for lithium on the RDP was observed at pH 10. This behavior of lithium ions adsorption is common due to the fact that at a low pH of 2, the solution and the adsorbent surface are highly protonated with H^+^, which competes with the protonated lithium ions for the available active sites on the RDP. This results in the lower adsorption removal for lithium at pH 2 compared to higher pH values. The adsorption removal for lithium on the RDP continued to increase from pH 4 (47.8%) and 6 (51%) as the concentration of H^+^ and its competing behavior decreased in the solution. The electrostatic attraction forces between the less protonated adsorbent functional groups and the protonated lithium-ion increased, which enhanced the adsorption removal. Moreover, the adsorption removal slightly increased as the pH increased from 8 (52.2%) to 10 (52.6%). This could be explained by two possible scenarios. Firstly, at pH 8, the solution became less concentrated with H^+^ and more concentrated with OH^-^. Therefore, the electrostatic attraction forces between the negatively charged adsorbent and the positively charged lithium-ion increased with the increase of pH to 8 and 10. This led to an increase in the adsorption removal for lithium on the RDP at pH 8 and 10. The second explanation for the observed highest adsorption of lithium-ion on the RDP at pH 10 could be due to the lithium precipitation and the formation of lithium hydroxide in the solution. This behavior corresponds to a faked removal behavior for metals at highly basic pH values^26^. A recent study done by Kamran and Park revealed that lithium removal onto a variety of acid functionalized carbon nanofibers decorated with Mn-doped TNT-nanocomposites favored increasing pH from 2 to 14. They discussed the effect of H^+^ concentration in the solution on decreasing the adsorption removal at low pH ranges^27^. Marthi and smith performed a study to compare the adsorption capacity of H_2_TiO_3_-diatomaceous earth composite towards lithium ions in brine and LiCl buffered solution. The results of the study revealed that lithium adsorption was enhanced by increasing the pH. Interestingly, lithium adsorption from the buffered solution was much greater than the brine solution. The reason for that is due to the lower pH value of the brine (pH 7.5) solution and the presence of other competing ions compared to the LiCl buffer solution (pH 9.5)^28^.

For RDP-FC-Ni, Fig. 1 shows a slight increase from pH 2 (56.1%) to 4 (56.2%) then a decrease at pH 6 (55.9%) followed by increases at pH 8 (56.1%) and 10 (57.8%) (*P* value < 0.05). The slight increase in the adsorption removal of lithium at pH 4 compared to pH 2 could be attributed to the same explanation mentioned earlier. However, it is important to note that, pH 4 is still acidic and the concentration of H^+^ is relatively high, which explains the slight increase in the adsorption removal from pH 2 to 4. The decrease in the adsorption removal for lithium at pH 6 could be explained by the decrease in the available active sites on the surface of RDP-FC-Ni as the adsorption reaches equilibrium. Furthermore, the gradual increase of the adsorption removal for lithium at the basic pH’s 8 and 10 is mostly due to the formation of lithium hydroxide as the concentration of OH^-^ in the solution increased^29^.

For RDP-FC-Cu, higher adsorption removals are observed at all pH values than RDP-FC-Ni and RDP (*P* value < 0.05). The possible reason could be due to the fact that copper has a higher molecular weight than nickel. This makes copper take a larger space in the formed metal hexacyanoferrate complexation. Compared to copper, lithium has a much smaller atomic weight. The atomic weight of lithium is equal to 6.941 g/mol while for copper it is 63.543 g/mol. The difference in the atomic weight of lithium and copper is much higher than with nickel (58.693 g/mol). This results in the higher affinity of lithium ions to take up the smaller space left in the copper hexacyanoferrate complex to form a more stable metal complexation than with the bigger space in the nickel hexacyanoferrate complex^30^.

According to the discussed results for the adsorption of lithium onto RDP as well as RDP-FC-Cu and RDP-FC-Ni at different pH values, the pH value was found to be effective in achieving the highest removal, and for industrial use is pH 6. The pH 6 was chosen to be the optimum pH value for the next experiments and the recovery for lithium ions because preparing it does not require the addition of high amounts of HCl. Furthermore, the previous results for the characterization of the collected reverse osmosis brine collected revealed that the pH was 7.8. This pH lies between 6 and 8, which showed relatively high adsorption removals for lithium in the study. Therefore, the recovery of valuable metals from the real reverse osmosis brine stream involved in the study could be implemented without any pH modifications. Indeed, this would be the most environmentally safe and economically friendly approach.

#### The zeta potential analysis for the roasted date pits and composites

**Figure 2:**
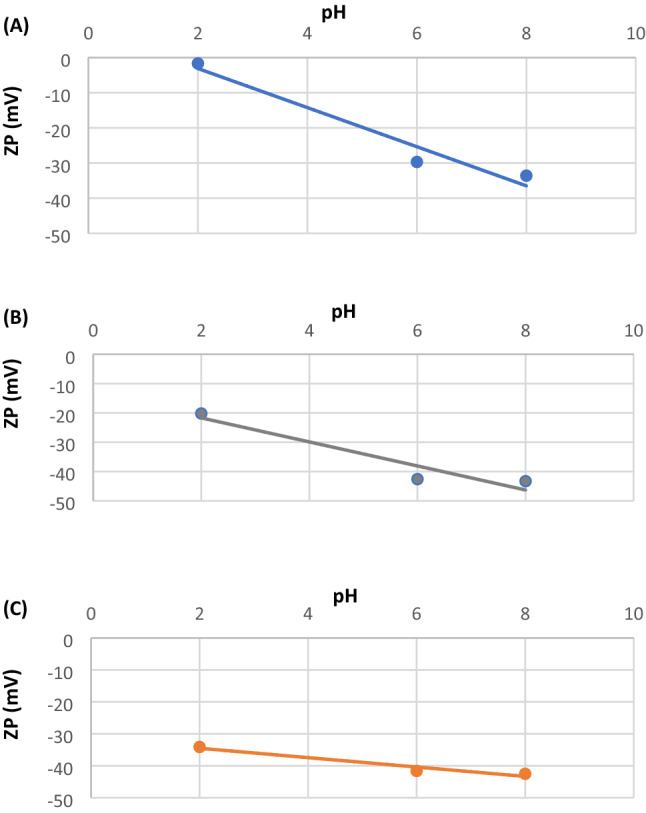
The zeta potential (ZP) analysis of (**A**) RDP, (**B**) RDP-FC-Cu, and (**C**) RDP-FC-Ni at pH of 2, 6, and 8. The experimental conditions were as follows: temperature of 25 ℃, 50 mL distilled water, around 0.05 g of adsorbent, and shaking time of 24 h. at 160 rpm.

The zeta potential (ZP) test was performed for the three adsorbents to study various aspects of the effect of solution pH on the adsorption of lithium onto the adsorbents. The pH ranges of the zeta potential test were 2, 6, and 8. The results are presented in Fig. 2A-C. For RDP (Fig. 2A), the zeta potential was found to be equal to − 1.67 mV, − 29.7 mV, and  − 33.6 mV for pH of 2, 6, and 8, respectively. The negative values indicate that the charges in the interface between the solid adsorbent and the liquid medium that contains lithium are negative, which poses electrostatic attraction forces that support the adsorption process. It can be noticed that the zeta potential values for RDP increase with an increase in pH. The zeta potential value obtained for RDP at pH of 6 is relatively highly negative ( − 29.7 mV); representing the stability of the adsorbent’s particles as well as their high adsorptive capabilities for lithium due to opposite charges and electrostatic attraction forces^31^. The zeta potential values obtained for RDP-FC-Cu are presented in Fig. 2B. Similar to RDP, the zeta potential values appear to negatively increase with an increase in pH from 2 to 8. However, the zeta potential values for RDP-FC-Cu are more negative than the zeta potential values for RDP. This means that RDP-FC-Cu is more stable in the adsorption solution than RDP and more electrostatically negative, which poses more electrostatic attraction forces towards the positively charged lithium ions^32^. This is supported by the fact that RDP-FC-Cu achieved higher adsorption removals than RDP at all pH values, and not just 2, 6, and 8. Moreover, RDP-FC-Ni showed similar results to RDP and RDP-FC-Cu in terms of increasing negativity of zeta potential with raising pH. Furthermore, the zeta potential values of RDP-FC-Ni (Fig. 2C) are closer to the values obtained for RDP-FC-Cu than RDP. However, at the optimum adsorption pH value of 6, the zeta potential value for RDP-FC-Cu was slightly higher than for RDP-FC-Ni, which shows higher stability for RDP-FC-Cu at that pH. The similarity in the zeta potential for both composites could be due to more common adsorbent characteristics between them than with the initial material (RDP). The high stability of RDP-FC-Ni is shown clearly from the overall highly negative zeta potential values obtained^33^. This indicates and further confirms previous results where RDP-FC-Ni showed closer adsorptive effectiveness to RDP-FC-Cu but higher than RDP for the adsorption of lithium.

### Effect of lithium concentration on the adsorption onto roasted date pits and two composites

**Figure 3:**
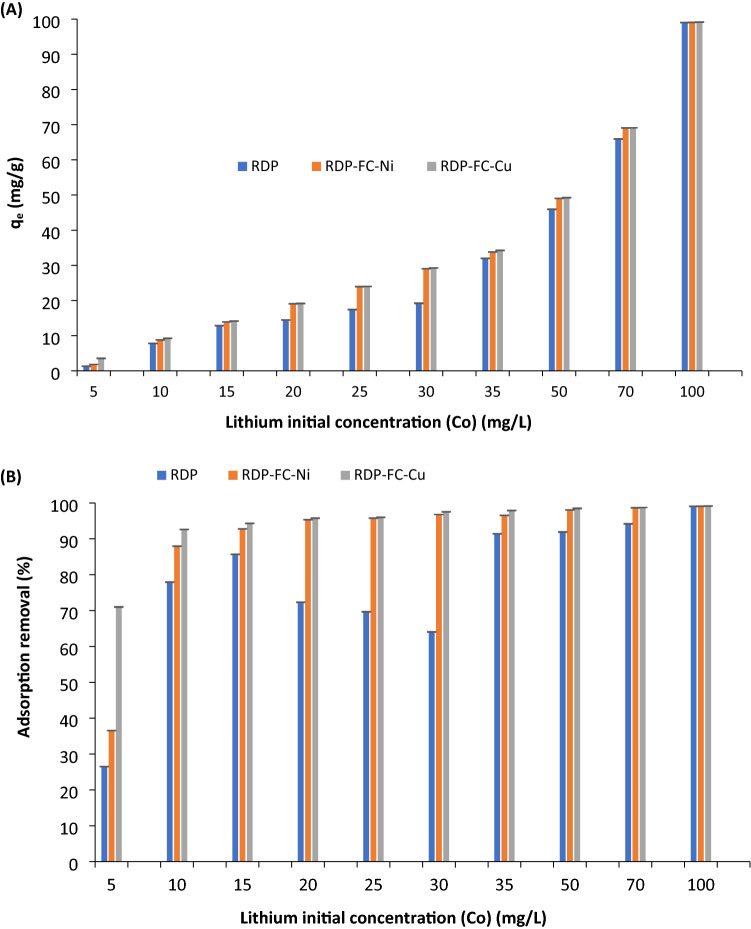
The effect of initial lithium concentration on (**A**) adsorption capacity and (**B**) adsorption removal (%) onto RDP, RDP-FC-Cu, and RDP-FC-Ni. The experimental conditions were as follows: the lithium concentrations studied were 5, 10, 15, 20, 25, 30, 35, 50, 70, and 100 mg/L. The pH was kept at the optimum pH of 6, the temperature of 25 ℃, shaking time of 24 h. at 160 rpm, and the adsorbent dose of 0.05 g. Error bars are shown in the Figure.

Figure 3A,B show the adsorption capacities (mg/g) and removals in terms of percentage for lithium onto RDP, RDP-FC-Cu, and RDP-FC-Ni at a variety of lithium concentrations. The lithium concentrations studied were (5, 10, 15, 20, 25, 30, 35, 50, 70 and 100) mg/L. The pH was kept at the optimum pH of 6, the temperature of 25 ℃, shaking time of 24 h, at 160 rpm, and adsorbent dose of 0.05 g.

As a general trend, Fig. 3 shows a constant increase in adsorption capacity with increasing lithium concentration for all adsorbents. It is evident that the adsorption capacity of RDP-FC-Cu towards lithium is similar to RDP-FC-Ni while it is the lowest for RDP, especially at lithium concentrations below 100 mg/L. This result is expected as it was shown previously that the adsorption removal of lithium at a variety of pH ranges was the highest for RDP-FC-Cu followed by RDP-FC-Ni and RDP. Moreover, it can be noticed as a general trend from Fig. 3A that the adsorption capacities of lithium onto RDP-FC-Cu and RDP-FC-Ni become very similar at lithium concentrations above 5 mg/L than to RDP. The adsorption capacities for the adsorption of lithium onto RDP were found to be 1.3 mg/g, 7.8 mg/g, 13 mg/g, 14.5 mg/g, 17.4 mg/g, 19.2 mg/g, 32 mg/g, 46 mg/g, 66 mg/g, and 99 mg/g for lithium concentrations of (5, 10, 15, 20, 25, 30, 35, 50, 70, and 100) mg/L, respectively (*P* value < 0.05). The RDP at study had many exceptional functional groups through the FTIR analysis that were involved in the adsorption of lithium ions at a variety of concentrations. The SEM analysis showed the smooth and porous surface morphology of RDP, which gave insights regarding the availability of sufficient amounts of adsorption active sites. The results of this experiment are similar, where the adsorption of lithium was found to continuously increase with an increase in the initial lithium concentration in the solution. The adsorption capacity of lithium onto RDP (Fig. 3A was found to be as high as 99 mg/g for an initial lithium concentration of 100 mg/L. Moreover, the adsorption of lithium onto RDP showed relatively close adsorption capacities for lithium concentrations of 5 mg/L (1.3 mg/g), 10 mg/L (7.8 mg/g), 15 mg/L (13 mg/g), 20 mg/L (14.5 mg/g), 25 mg/L (17.4 mg/g) and 30 mg/L (19.2 mg/g). The adsorption capacity can be seen to increase at a higher rate from 19.2 to 32 mg/g when the initial lithium concentration increased from 30 to 35 mg/L, respectively. At lithium-ion concentrations of 35 mg/L and higher (50 mg/L, 70 mg/L, and 100 mg/L), the adsorption capacities of RDP maintained the same trend of highly increasing. At 50 mg/L lithium concentration, the adsorption capacity was observed to be 46 mg/g while at 70 mg/L and 100 mg/L lithium concentration, the adsorption capacities were 66 mg/g and 99 mg/g, respectively. The differences in the adsorption capacities between each successive initial lithium concentration are illustrated in Table 1. It is clear from the table that the adsorption capacity increased at a constant and slower pace at lower lithium concentrations than 35 mg/L and higher.

**Table 1:** The differences in the adsorption capacities of lithium onto RDP at a variety of initial lithium concentrations.

Initial lithium concentration (mg/L)	Difference in adsorption capacity (q_e_) (mg/g)
5 and 10	6.5
10 and 15	5.2
15 and 20	1.5
20 and 25	2.9
25 and 30	1.8
30 and 35	12.8
35 and 50	14
50 and 70	20
70 and 100	33

The trend observed in Table 1 can be explained by the following: at low lithium concentrations, the ratio of the number of the available adsorption active sites to lithium concentration is high. This means that there are plenty of vacant sites for the low lithium ions to bind. This facilitates the adsorption process due to the great functionality, structural morphology, and affinity of RDP towards the adsorption of lithium ions. The slower pace of increased adsorption observed for initial lithium concentrations below 35 mg/L could be due to less mass transfer and collision between the lithium species and the binding sites^34,35^. The continuous increase in the adsorption capacity for lithium with increasing initial lithium concentrations could be mainly attributed the increased lithium mass transfer onto the available adsorption active sites on the RDP surface^36^. However, it is expected that at initial lithium concentrations above 100 mg/L the adsorption capacity to reach an equilibrium status and a maximum removal state. This is because the adsorption removal and capacity of lithium reached almost 100% (99 mg/g q_e_ and 99% adsorption removal) at an initial lithium concentration of 100 mg/L. The equilibrium and maximum pollutant removal phenomena are common in adsorption systems. This is because as the lithium concentration increases, the mass transfer would increase its adsorption onto the available active sites. However, the available active sites on any adsorbent are finite. Therefore, excess lithium ions would remain in the solution and the adsorption capacity would reach a plateau^34,37^. Several studies reported similar results where Al-Ghouti and others investigated the effect of mercury solution concentration on its adsorption capacity onto roasted date pits. The results revealed that the adsorption of mercury increased with increasing concentration due to the availability of adsorptive active sites as well as enhanced mass transfer forces. However, a constant adsorption behavior was observed for higher mercury concentrations because of the filling of the vacant sites^17^. Samra reported that metals could have enhanced adsorption onto roasted date pits with increased concentrations because of increased metal diffusion processes onto the adsorbents^38^. Furthermore, Al-Ghouti and others reported that the enhanced adsorption capacity of bromide ions with increased concentration is a predictable adsorption behavior. This was mainly due to the great collision and mass transfer forces between the adsorbent and the metal. The diffusion of bromide into the boundary layer of the adsorbent was enhanced when the bromide concentration was elevated per unit weight of the roasted date pits.

The adsorption of lithium onto RDP-FC-Ni and RDP-FC-Cu follows the same increasing trend with lithium concentration as RDP (Fig. 3A,B). This indicates the consistency of the adsorption patterns and favorability to increasing concentrations despite the surface modifications on the RDP. The differences in the adsorption in terms of percentages are significant between the three adsorbents (*P* value < 0.05). It is worth noting that the adsorption capacity of RDP-FC-Cu was very similar to RDP-FC-Ni especially at higher initial lithium concentrations. In fact, the adsorption capacities obtained with the adsorption of lithium onto RDP-FC-Cu were (3.55, 9.26, 14.15, 19.15, 24, 29.26, 34.26, 49.25, 69.11, and 99.15) mg/g for initial lithium concentrations of (5, 10, 15, 20, 25, 30, 35, 50, 70 and 100) mg/L, respectively (*P* value < 0.05). To compare, the achieved adsorption capacities with the adsorption of lithium onto RDP-FC-Ni are (1.83, 8.79, 13.92, 19.07, 23.94, 29.03, 33.78, 49.03, 69.07, and 99.08) mg/g (*P* value < 0.05). Despite the enhanced morphological structure in terms of surface area, pore radius, and volumes as well as and cavities for RDP when compared to raw-DP, the prepared composites involved in the study showed enhanced surface area, pores radius, and volume, and cavities. This could indicate the significant role of the higher surface area and adsorption active sites on the prepared composites than the RDP. The illustrated BET surface area analysis confirmed the increase in the total surface area for adsorption of the prepared composites from around 2.5 m^2^/g for RDP to 4.7 m^2^/g for RDP-FC-Cu and 5.2 m^2^/g for RDP-FC-Ni. It can also be noticed that the prepared composites resulted in similar adsorption capacities and removals towards lithium at a variety of initial concentrations when compared to RDP (Fig. 3A,B). This could be because the prepared composites have more characteristic functional groups, structure, and composition in common that played significant roles in the adsorption process when compared with their starting material (RDP). This was shown in the FTIR results where the characteristic functional groups like C≡N and Fe–C showed altered peaks due to lithium adsorption**.** Furthermore, similar trends are observed with RDP-FC-Cu and RDP-FC-Ni as with RDP in terms of a sudden increase in the adsorption capacity towards lithium at concentrations above 35 mg/L. Moreover, it is worth noting that the adsorption capacity of lithium onto RDP, RDP-FC-Cu, and RDP-FC-Ni at an initial lithium concentration of 100 mg/L is almost the same (99.04 mg/g for RDP, 99.15 mg/g for RDP-FC-Cu, and 99.08 mg/g for RDP-FC-Ni).

As mentioned earlier, the higher adsorption capacities observed for lithium at concentrations of 35 mg/L and higher for all adsorbents could be mainly due to mass transfer forces. This could be justified by the fact that lithium ions in aqueous solutions have an ionic radius of 3.4 Å. Usually, in metal adsorption systems, alkali metals with the smallest ionic radius achieve the highest adsorption capacities onto adsorbents due to the shorter distance between the metal and the adsorbent’s surface^35^. In the case of lithium adsorption onto the adsorbents at higher concentrations, the lithium ions are closer to each other, and the adsorbents surface. This results in more collisions and confirms that mass transfer played a significant role in the sudden increases in the adsorption capacities.

The high adsorptive capacity of RDP-FC-Cu and RDP-FC-Ni towards lithium than RDP could be due to multiple reasons. Firstly, this adsorption behavior was more notable at high lithium initial concentrations (higher than 5 mg/L). At low initial lithium concentration (5 mg/L), the adsorption binding active sites at the three adsorbents are plenty. The ratio of the binding sites and lithium concentration is high; therefore, the pore filling is favorable. At higher lithium initial concentrations, the ratio of the adsorption binding sites to the lithium concentration is lower; therefore, the composites showed similar adsorption capacities due to their similar and enhanced morphological and chemical characteristics. Another reason could be due to the fact that copper has a hydrated ionic radius of 4.19 Å, which is close to the ionic radius of lithium ions (3.4 Å). On the other hand, the ionic radius of nickel is equal to 2 Å. This could mean that during the adsorption process, higher ion exchange, and adsorption, occurred between copper followed by nickel present in the potassium hexacyanoferrate and the lithium present in the solution. This usually takes place when the target metal could replace the existing metal present in the potassium metal hexacyanoferrate due to a similar ionic radius. The somewhat similar hydrated ionic radius of lithium, copper, and nickel, explains the similar adsorption capacities between RDP-FC-Cu and RDP-FC-Ni towards lithium. Another possible adsorption mechanism that resulted in the more favorable adsorption of lithium onto RDP-FC-Cu and RDP-FC-Ni is ion exchange with potassium present in the complex. This forms a stable metal hexacyanoferrate complexation. Potassium has a hydrated ionic radius of 3.31 Å, which is very close to the hydrated ionic radius of lithium (3.4 Å), resulting in a more favorable ionic substitution mechanism into the metal coordination cubic complexes^39^.

The slightly higher adsorption capacity of RDP-FC-Cu towards lithium when compared to RDP and RDP-FC-Ni could be due to other characteristics of the adsorbent that enhanced its selectivity towards lithium (Fig. 3A). The size of the hydrated radius of lithium and copper is more similar than that of lithium and nickel, which explains the favorability of ionic exchange between lithium and copper in the metal complex. The BET results show that the surface area of RDP-FC-Cu was enhanced compared to RDP. To be exact, the surface area of RDP-FC-Cu is around 4.6 m^2^/g. The pore radius of RDP-FC-Cu is equal to 138.6 Å while it is equal to 39.2 Å for RDP-FC-Ni. Moreover, the pore volume of RDP-FC-Cu is higher than the pore volume of RDP-FC-Ni, which provided more and deeper adsorption active binding sites for lithium. These adsorption behaviors of metals onto potassium hexacyanoferrates have been reported by many studies. Loos-Neskovic and others reported that the adsorption of cesium onto potassium copper hexacyanoferrate resulted in the release of potassium ions in the solution as a consequence of cesium adherence onto the adsorbent’s surface. The authors mentioned that copper was also released in the solution, however at much less concentrations than potassium^40^. Interestingly, the ionic radius of cesium is equal to 2.26 Å, which is closer to the ionic radius of potassium (3.31 Å) than copper (4.19 Å). This facilitated the ionic exchange of cesium with potassium at a higher quantity than with copper. Another study was performed by Naidu and others on the adsorption of alkali metals onto laboratory synthesized and commercial potassium cobalt hexacyanoferrate adsorbent. The results of the study revealed that both adsorbents achieved higher adsorption capacities towards rubidium than cesium. This is due to the occurrence of a true ion exchange occurs between the potassium and rubidium. The authors reported that the adsorbent’s cavity sizes are more similar to the rubidium radius than cesium, which resulted in a greater penetration of rubidium into the adsorbent’s lattice. Consequently, the replacement of potassium takes place, and a higher adsorption capacity is observed. The authors also discussed that cobalt and iron were released in minute amounts into the solution, which indicates that the adsorption of the metals occurred mainly with the displacement of potassium. This is because transition metals are usually bound through strong cyano-groups bridges, which provide the hexacyanoferrate lattice with a balanced negative charge and stability^41^. Moreover, the adsorption of cesium onto a potassium nickel hexacyanoferrate adsorbent was investigated by Michel and others. The results showed that the adsorption of cesium favored the exchange with potassium more than nickel in the crystal lattice. It was reported that the amount of adsorbed cesium was more similar to the amount of potassium released than nickel. The potassium and cesium exchange proceeded at 80%, while the nickel and cesium exchange proceeded at 20%, where two ions of cesium replaced one ion of nickel in the crystal lattice^42^.

### Physiochemical characterization of the prepared adsorbents

#### Scanning electron microscopy (SEM)

**Figure 4:**
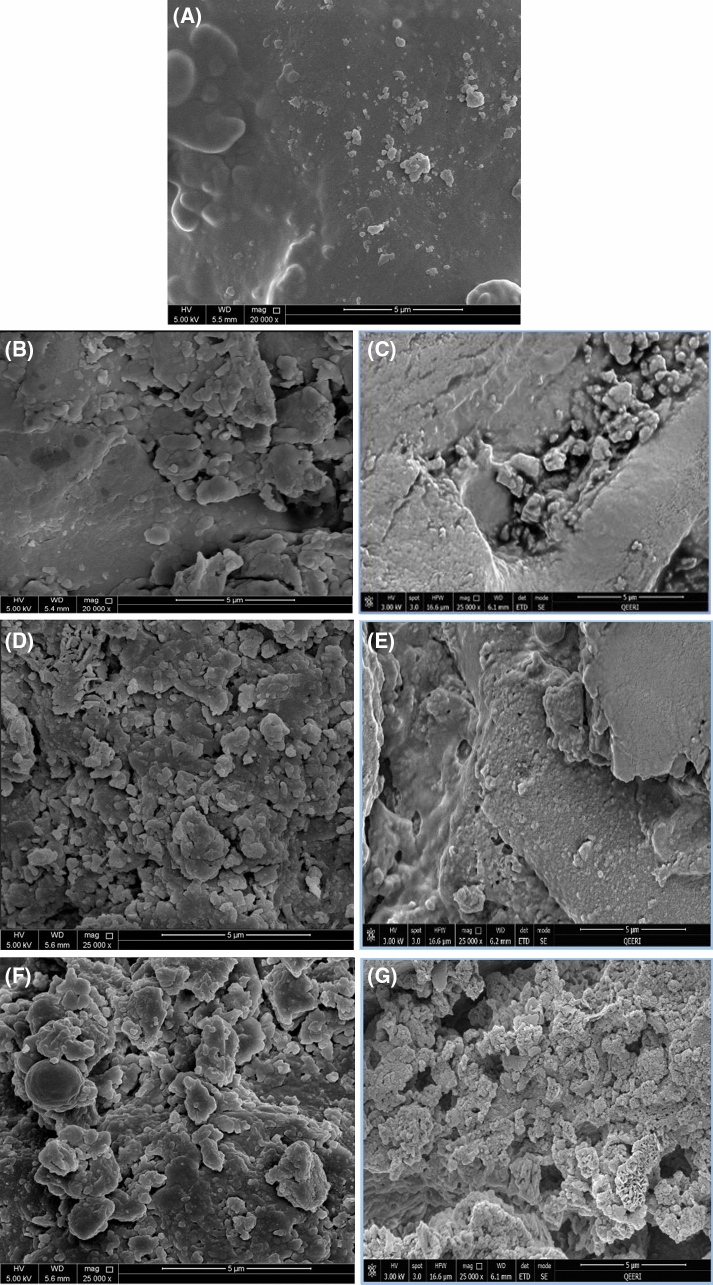
The SEM morphological images of (**A**) raw-DP (20,000 ×), (**B**) RDP before lithium adsorption (20,000 ×), (**C**) RDP after lithium adsorption (25,000 ×), (**D**) RDP-FC-Ni before lithium adsorption (25,000 ×), (**E**) RDP-FC-Ni after lithium adsorption (25,000 ×), (**F**) RDP-FC-Cu before lithium adsorption (25,000 ×), and (**G**) RDP-FC-Cu after lithium adsorption (25,000 ×).The after the lithium adsorption conditions are 45℃, 50 mL volume, 100 mg/L lithium concentration, shaking time of 24 h at 160 rpm, and 0.05 g adsorbent mass.

Figure 4A-G shows the SEM images for the raw-DP as well as the three adsorbents before and after the adsorption of lithium. As can be seen from Fig. 4A, the raw-DP before roasting showed a smooth somewhat flattened surface with few scattered small-sized elevations. To compare, the RDP pits before the adsorption of lithium (Fig. 4B) demonstrate a rugged dense mass structure with elevated formations of varying shapes and sizes and visible cavities throughout. It was shown that the adsorption capacity of the adsorbents increased significantly with an increase in temperature from 25 to 45 ℃. The great morphological change can be seen on the RDP after the adsorption of lithium (Fig. 4C) at the temperature that achieved the highest adsorption capacity (45 ℃). The adsorption of lithium onto the RDP along with the working temperature of 45 ℃ led to the formation of more pronounced similar-sized protrusions on the surface. This could be due to the high temperature, which resulted in the cracking of the surface as well as the accumulation of lithium ions on the surface of RDP. RDP-FC-Ni before the adsorption of lithium (Fig. 4D) appears to have smaller formations, pores, and cavities than RDP-FC-Cu (Fig. 4F). After the adsorption of lithium, RDP-FC-Ni (Fig. 4E) showed significant morphological change where the surface appeared to be more flattened, which indicates the filling of the available active sites (pores) of the adsorbent. For RDP-FC-Cu (Fig. 4G), the adsorption of lithium has also led to changes in the morphology in terms of the formation of more dense smaller-sized pronounced protrusions^43,44^.

#### Particle size distribution (PSD)

The particle size distribution test presents the full distribution pattern of the different particle sizes present in a homogenous sample of an adsorbent. Through this technique, one can view the different particle sizes a sample holds in different proportions in terms of percentage. Thus, this technique is based on the assumption that the total amount of particles in a sample is 100%^45^. Table 2 represents the D10, D50, and D90 for the RDP, RDP-FC-Cu, and RDP-FC-Ni. The D10 shows the average size of 10% of a given adsorbent while the D50 and D90 represent the average sizes of 50% and 90% of the adsorbent, respectively. From Table 2, it can be noticed that the largest D10, D50, and D90 are achieved for RDP while the smallest values are obtained for RDP-FC-Ni. For RDP, 10% of the sample has a particle size less than 33.9 µm, 50% of the sample is smaller than 751.1 µm and 90% of the sample is smaller than 1,159.6 µm. The RDP-FC-Cu showed particles smaller than 29.1 µm for 10% of the sample, while 50% of the sample was less than 195.5 µm and 90% of the sample’s particle size was less than 906 µm. Interestingly, the largest portion of the RDP-FC-Cu sample (90%) was below 1000 µm while the largest portion of the RDP was still in the range of 1000 µm. This means that most of the particles of the RDP-FC-Cu are smaller than most of the particles of RDP. Moreover, 10% of the RDP-FC-Ni sample has particle sizes less than 1 µm while 50% of the sample showed particle sizes less than 26.6 µm and 90% of the sample had particle sizes less than 804.3 µm. These results indicate that the majority of the RDP-FC-Ni sample had particle sizes less than the other prepared composite (RDP-FC-Cu) and the initial adsorbent material (RDP). These results are predictable as the preparation process of the prepared composites involved the addition of potassium hexacyanoferrate, sodium hydroxide, strong stirring, centrifuging, and heating in the oven. Not all these steps were done for the simple preparation of the RDP. Moreover, the addition of the potassium hexacyanoferrate could have led to a decrease in the particle sizes of the composites due to alteration of the structures (formation of cubic crystal lattices), and breakage of particles. It is well established that sodium hydroxide is a strong base, which may lead to the reduction of powder-like materials like RDP. Koutu and others demonstrated that the addition of NaOH to zinc oxide nanoparticles resulted in the subsequent decrease in their particle size distributions^46^. The smaller particle size of the RDP-FC-Ni compared to RDP-FC-Cu could be attributed to the fact that the atomic mass and radius of nickel are smaller than the atomic mass and radius of copper. The atomic mass and radius of nickel are 58.693 pm and 124 pm (covalent radius), respectively. On the other hand, the atomic mass and radius of copper are 63.546 pm and 124 pm, respectively. This leads to less space occupied for nickel in the potassium hexacyanoferrate lattice than copper and ultimately, the smaller particle size of the composite adsorbent.

**Table 2:** Particle size distribution of RDP, RDP-FC-Cu, and RDP-FC-Ni.

Adsorbent	D10	D50	D90
RDP	33.9	751.1	1159.6
RDP-FC-Cu	29.1	195.5	906
RDP-FC-Ni	1	26.6	804.3

#### Brunauer–Emmett–Teller (BET) surface area, pore size, and volume distribution

The BET surface area, pore size, and volume distribution tests were conducted for the RDP, RDP-FC-Cu, and RDP-FC-Ni, and the results are presented in Table 3. The results demonstrate the increase in the surface area upon the modification of RDP into RDP-FC-Cu and RDP-FC-Ni. The surface area of RDP-FC-Ni was found to be the highest (5.262 m^2^/g) but close to the value obtained for RDP-FC-Cu (4.758 m^2^/g). Moreover, the pore size distribution shows that RDP-FC-Cu obtained the highest pore size of 138.6 Å compared to 82 Å for RDP-FC-Ni and 39.2 Å for the RDP. Interestingly, there was a slight difference between the pore volumes of RDP and RDP-FC-Ni while RDP-FC-Cu showed the largest pore volume distribution of around 0.033 cubic centimeters per gram.

**Table 3:** BET surface area, pore size, and volume distribution test of RDP, RDP-FC-Cu, and RDP-FC-Ni.

Adsorbent	Surface area (m^2^/g)	Pore size (Å)	Pore volume (cc/g)
RDP	2.518	39.2	0.010325
RDP-FC-Cu	4.758	138.6	0.032964
RDP-FC-Ni	5.262	82.0	0.010306

#### Carbon and nitrogen elemental analysis

The chemical composition of the raw-DP, RDP, RDP-FC-Cu, and RDP-FC-Ni are presented in Table 4. It is clear from the results that the carbon content of the materials is increasing upon roasting and modification using potassium metal hexacyanoferrates. On the other hand, the nitrogen content decreased upon roasting of the date pits and modifications. The enhanced carbon contents could be due to the addition of hexacyanoferrates, heating, and roasting. The loss of nitrogen upon roasting and modification could be attributed to the fact that roasting and heating usually lead to the loss of nitrogen gas^47^.

**Table 4:** The carbon and nitrogen elemental analysis of raw-DP, RDP, RDP-FC-Cu, and RDP-FC-Ni.

Material/Adsorbent	Carbon (%)	Nitrogen (%)
Raw-DP	36.12	7.32
RDP	37.42	5.04
RDP-FC-Cu	45.75	1.1
RDP-FC-Ni	46.04	1.07

#### X-ray diffraction (XRD) analysis

**Figure 5:**
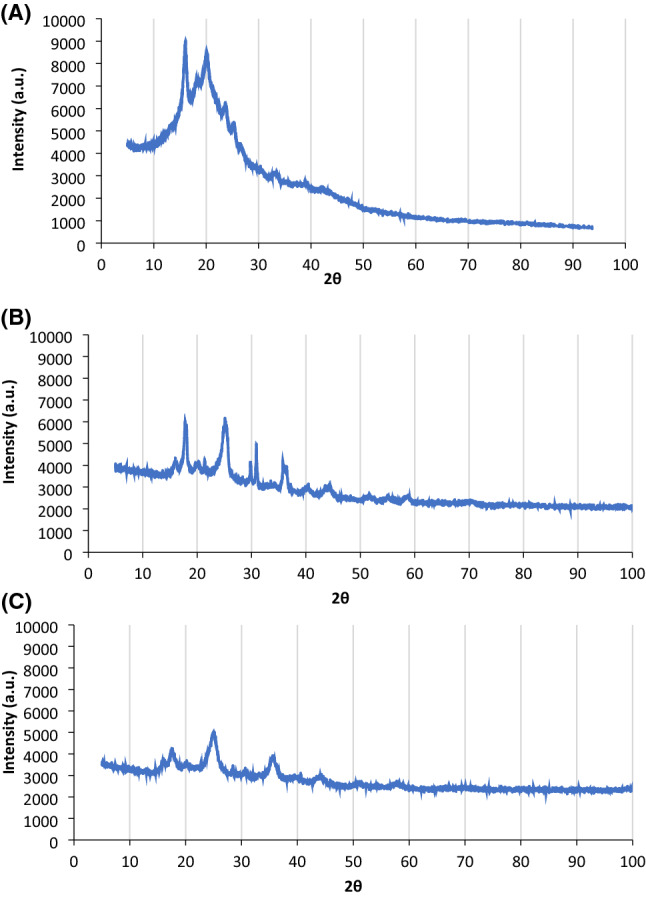
The XRD peak results of (**A**) RDP, (**B**) RDP-FC-Cu, and (**C**) RDP-FC-Ni.

Figure 5A-C represents the XRD of RDP, RDP-FC-Cu, and RDP-FC-Ni before the adsorption of lithium. From a general perspective, it can be noticed that the XRD of RDP (Fig. 5A) differs from the XRD’s of the prepared composites in Fig. 5 (RDP-FC-Cu (B) and RDP-FC-Ni (C)), while the composites obtained more similarities in the XRD trend and peaks. For RDP (Fig. 5A), there are two major intense peaks at 2θ 16° and 20°. The peak obtained for RDP at 2θ 16° is more intense (intensity of 9014) than the peak obtained at 2θ 20° (intensity of 8605). This means that at the 2θ 16° spacing between the atoms of the adsorbent, the crystals of the sample were more aggregated together than at the 2θ 20°. Moreover, for RDP, the peak at 2θ 16° is narrower than the peak at 2θ 20°. This means that at 2θ 16°, the crystals of the samples are bigger in size than the ones obtained at 2θ 20° phase or spacing^48^. For RDP-FC-Cu (Fig. 5B), the XRD shows 4 distinctive sharp and intense peaks at 2θ 17°, 25°, 30°, and 35°. These peaks indicate the high crystallinity of the sample along with the presence of smaller size crystals than RDP. In particular, it appears that the crystals in the RDP-FC-Cu are similar in size. In addition, the XRD peaks were similar to other studies that reported that the structure is an F-centered cubic unit cell^49,50^. The XRD peaks obtained for RDP-FC-Cu were significantly different from the XRD peaks obtained for RDP. This shows that the modification done to the RDP to prepare the RDP-FC-Cu was effective. Interestingly, the peaks obtained for RDP-FC-Cu were consistent with other studies that involved copper-based hexacyanoferrates^20,36^. The XRD pattern obtained for RDP-FC-Ni (Fig. 5C) shows the same peaks that were obtained for RDP-FC-Cu. This shows that both composites have similar crystalline and structural characteristics and that the modification was successful. However, a peak at 2θ 30° disappeared for the RDP-FC-Ni adsorbent, which, according to studies, was found to be a characteristic peak for copper potassium hexacyanoferrate composites^48^.

#### Fourier-transform infrared spectroscopy (FTIR) analysis

**Figure 6:**
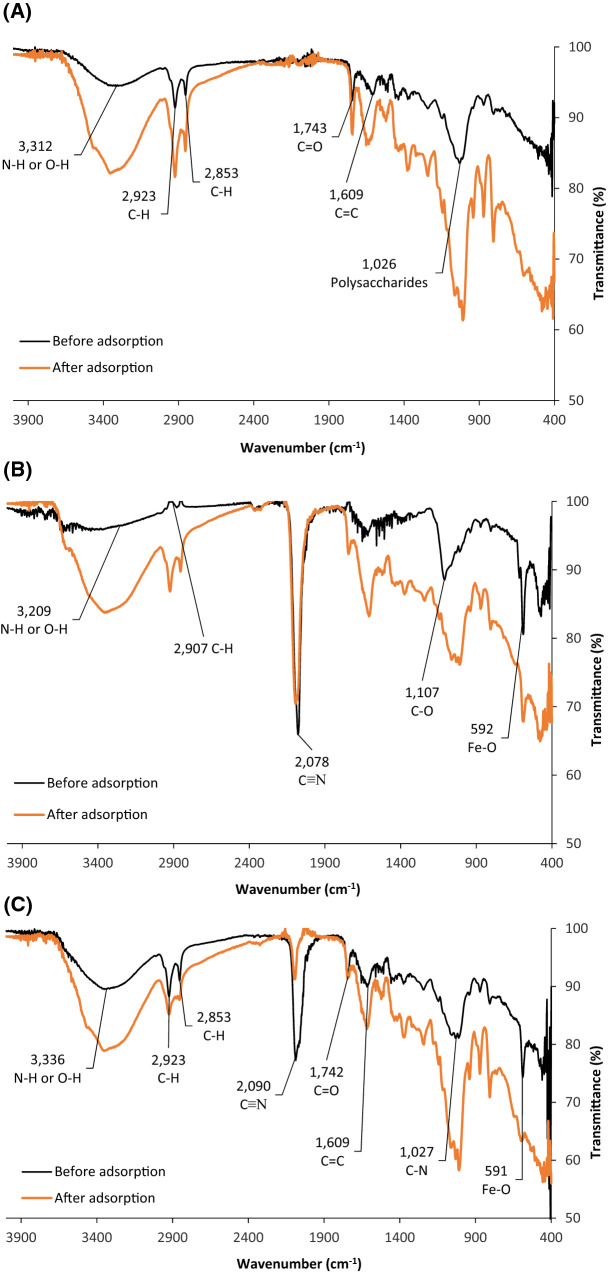
(**A**) FTIR spectra before and after the adsorption of lithium onto RDP, (**B**) RDP-FC-Cu, and (**C**) RDP-FC-Ni. The experimental conditions were as follows: temperature of 45 ℃, 50 mL volume, 100 mg/L lithium ions concentration, shaking time of 24 h. at 160 rpm, and 0.05 g.

Figure 6A-C shows the FTIR spectrum for the three adsorbents before and after the adsorption of lithium. The results show that the RDP (Fig. 6A) is more similar to RDP-FC-Ni (Fig. 6C) in terms of functional groups than to RDP-FC-Cu (Fig. 6B). RDP-FC-Cu shows the least number of functional groups compared to the RDP and RDP-FC-Ni composite. For RDP before adsorption, the medium peak at around 3312 cm^−1^ corresponds to the O–H bond or N–H stretching (secondary amine). The medium peaks at 2923 cm^−1^ and 2853 cm^−1^ are for C-H stretching vibrations such as CHO, and CH_3_CH_2_- (ethyl molecules). In addition, the strong peak at 1743 cm^−1^ represents the C=O bond of esters^16,51^. The RDP contains a C=C stretching bond that represents conjugated alkenes at 1609 cm^−1^. Furthermore, the RDP demonstrated a peak at around 1026 cm^−1^, which belongs to polysaccharides mainly hemicelluloses, and functional groups like functional groups such as C–O, C–C, C–O–P, and C–O–C vibrations^34,52^. After the adsorption of lithium onto the RDP (Fig. 3A), all of the observed bands are seen to become more intense in terms of increased absorbance and decreased transmittance. This indicates that all of the mentioned functional and characteristic groups of RDP played significant roles in the uptake of lithium. The broad peak at 3312 cm^−1^ became less broad and more intense after lithium adsorption. Besides, the peak at around 1026 cm^−1^ had a slight shift to a wavenumber of around 1007 cm^−1^ upon the adsorption process. These changes in the FTIR peaks after the adsorption of lithium ions indicate that the specific molecules were involved directly and played a role in the adsorption process. In other words, the uptake of lithium ions by the adsorbent resulted in an altered composition of the adsorbent, which means that these functional groups are important to be present for the adsorption to be efficient^51–53^.

Moreover, the FTIR spectrum for the original RDP-FC-Cu composite (Fig. 6B) demonstrates the functional changes that occurred due to the modification of the RDP. These functional changes are represented by the broadening of the O–H or N–H stretching peak which is shown at 3209 cm^−1^^34,51^. Also, the peak that was present for the RDP at 2923 cm^−1^ (C–H bond) can be seen to become less intense and shifted for the RDP-FC-Cu (2907 cm^−1^) when compared to RDP. This could be due to the chemical change that occurred on the overall cellulosic mass of the RDP upon modifications. Moreover, the peaks that were present for RDP at 2853 cm^−1^, 1743 cm^−1^, 1609 cm^−1^, and 1026 cm^−1^ disappeared for the RDP-FC-Cu. This further confirms the chemical modification and change on the RDP. Furthermore, a strong sharp peak appeared for the RDP-FC-Cu at 2078 cm^−1^ which corresponds to the C≡N bond indicating the presence of K_4_[Fe(CN)_6_] and the modification by the potassium hexacyanoferrate^54^. Another characteristic functional group is shown on the FTIR spectrum of the RDP-FC-Cu at 1107 cm^−1^. This wavelength belongs to the C–O stretching bond of secondary alcohols^16^. The formation of a Fe–O bond due to the modification of the RDP into RDP-FC-Cu is shown by the peak at 592 cm^−1^, which confirms the presence of iron in the date pits. Major changes occurred to the FTIR bands of RDP-FC-Cu after the adsorption of lithium (Fig. 6B) where all the peaks, except for the characteristic peak of C≡N, became more intense (less transmittance and more absorbance). Similar to the results of RDP, this shows the significant chemical and structural changes that the adsorption of lithium-induced in the RDP-FC-Cu composite.

Furthermore, the FTIR spectrum for the original RDP-FC-Ni composite (Fig. 6C) shows the highest number of functional groups. This means that the modification of the RDP to RDP-FC-Ni mostly led to an enhancement of the functionality of the adsorbent. However, the RDP-FC-Ni still represents most of the functional groups found on the original roasted date pits. For example, the peak that corresponds to O–H or N–H bondage is less broadened (3336 cm^−1^) than the peak for RDP-FC-Cu^34,51^. The medium peaks that represent C-H stretching at 2923 cm^−1^ and 2853 cm^−1^ can be found for the RDP-FC-Ni^16^. Interestingly, the strong sharp peak that represents the C≡N bond and indicates the presence of K_4_[Fe(CN)_6_] modification, is also found for the RDP-FC-Ni as for the RDP-FC-Cu^34,54^. However, the peak is less sharp and strong as well as it can be seen at a slightly different wavelength of 2090 cm^−1^. The higher intensity of the C≡N peak for RDP-FC-Cu compared to RDP-FC-Ni is attributed to more concentration of C≡N bonds in the sample. Also, copper is highly electronegative, which gives rise to strong bonds. Additionally, copper has a higher atomic weight than nickel, which takes up a larger space in the formed metal hexacyanoferrate complexation. These factors result in the formation of strong and sharp peaks in an FTIR spectrum. The shift of the peak from a lower wavelength (2078 cm^−1^) for RDP-FC-Cu to a higher wavenumber (2090 cm^−1^) for RDP-FC-Ni is due to the fact that the mass of the complex is lower than RDP-FC-Cu. In FTIR principles, the mass of the vibrating molecule is inversely proportional to the frequency of vibration^30^. Moreover, the RDP-FC-Ni has C=O, C=C, polysaccharides, and Fe–O peaks at 1742 cm^−1^, 1609 cm^−1^, 1027 cm^−1^, 591 cm^−1^ respectively^16,17^. The adsorption of lithium onto the RDP-FC-Ni composite (Fig. 6C) has demonstrated similar FTIR results obtained for RDP-FC-Cu, which are discussed above (Fig. 6B).

Furthermore, similar results were obtained by other studies done on metal hexacyanoferrates. For example, Long and coworkers aimed at the synthesis of copper hexacyanoferrates nano-particles film for the recovery of cobalt. The functional groups for the adsorbent were determined using FTIR technology. The results revealed the presence of a C≡N bond through the appearance of an FTIR band at 2099 cm^−1^. It was found that the synthesized adsorbent had a Fe–C bond through the presence of bands at around 596 cm^−1^. These bonds illustrate and confirm further the modification of the nanoparticles by hexacyanoferrates^55^.

#### Thermogravimetric analysis (TGA)

**Figure 7:**
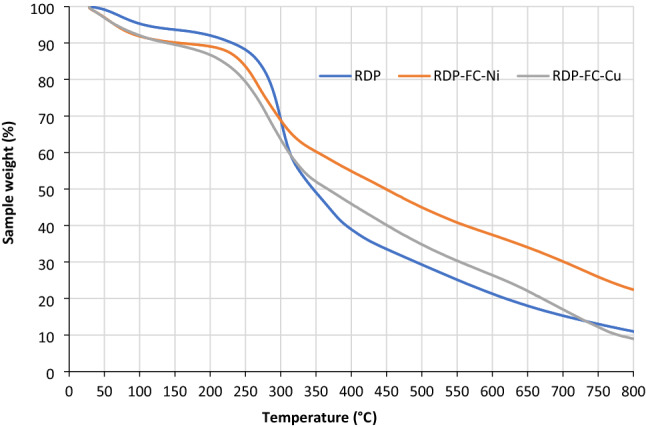
The TGA results of the RDP, RDP-FC-Cu, and RDP-FC-Ni.

The thermal stability characteristics of adsorbents can be identified precisely and accurately by performing a thermogravimetric (TGA) analysis. Therefore, the RDP, RDP-FC-Cu, and RDP-FC-Ni adsorbents were examined through a TGA analysis, and the results are shown in Fig. 7. The TGA results of the RDP reveal that an initial weight loss was observed to occur at a temperature of around 65 ℃. After a further increase of the temperature to around 250 ℃, the RDP experienced a somewhat constant weight loss. However, above the temperature of 250 ℃, a sharp followed by a gradual decrease in the weight of RDP took place. On the other hand, RDP-FC-Cu and RDP-FC-Ni obtained similar TGA results where they follow almost the same trend in their thermal decomposition with temperature. RDP-FC-Ni showed a gradual and minute decrease in its weight percentage at a temperature of around 37 ℃, followed by a stabilization trend (Constant weight) between 116 ℃ and 211 ℃. Above this temperature (211℃), gradual and constant degradation of the RDP-FC-Ni is observed. RDP-FC-Cu obtained a minute decrease in its weight percentage at around 38 ℃ followed by a stabilization in its weight between temperatures of 110 ℃ and 175 ℃. Above 175 ℃, gradual and constant degradation trends can be seen for RDP-FC-Cu with temperature. Interestingly, the constant thermal degradation observed for RDP-FC-Ni occurred at around 300 ℃, while for RDP-FC-Cu it took place at a higher temperature of around 344 ℃. The initial minute decreases in the weight percentages of the three adsorbents at lower temperatures could be due to the loss of moisture and volatile content. The following stages of weight loss can be attributed to the loss of celluloses, hemicelluloses, and main compounds, as well as the breaking of chemical bonds, and lastly carbonization^47^. From the results, it appears that RDP achieved moisture and volatile contents loss at a higher temperature than the prepared composites. This could be due to its rigid carbonaceous and lignocellulosic characteristics as well as the fact that no solvents were used to prepare the roasted date pits. It is more probable that as the prepared composites compromise a cubic lattice structure, it is much easier for moisture to evaporate at lower temperatures. Moreover, the almost constant degradation of RDP at a wide range of temperatures (65–250 ℃) corresponds to their possible prolonged thermal stability. The RDP-FC-Ni showed prolonged thermal stability of its weight between temperatures of 116 ℃ and 211 ℃, which is a wider range of temperatures than the stabilization trend that was observed for RDP-FC-Cu (temperatures between 110 ℃ and 175 ℃). However, the final stage of the thermal degradation of RDP-FC-Cu was noticed at higher temperatures than RDP-FC-Ni, which indicates its durability to a wider overall range of temperatures than RDP-FC-Ni. Overall, it can be noticed from the figures that the three adsorbents exhibit similar overall weight loss profiles, which confirms the modification of the RDP into the prepared composites without the complete loss of the initial characteristics. These results demonstrate the sufficient thermal stabilities and durability’s of the three adsorbents involved in this study.

### Desorption studies

The desorption of lithium and regeneration of the RDP, RDP-FC-Cu, and RDP-FC-Ni was studied. The ability to desorb lithium ions and regenerate the adsorbent material is of great importance in optimizing an environmentally friendly adsorption method. The study examined the effect of HCl concentration on the desorption of lithium by applying 0.5 M and 1 M HCl to the spent adsorbents (RDP, RDP-FC-Cu, and RDP-FC-Ni). The results of the study indicated that there was no significant difference between the achieved adsorption under 0.5 M and 1 M HCl (*P* value > 0.05). Also, the study revealed that the desorption of lithium was as high as 99% from all the adsorbents under the influence of both HCl concentrations. This indicates that lithium forms weak physical bonds with the adsorbents, which allows for their efficient regeneration and re-use. As a result, RDP, RDP-FC-Cu, and RDP-FC-Ni could be effectively used multiple times for the adsorption of lithium from RO brines.

### Competitive adsorption studies

As mentioned earlier, the presence of various metal ions in RO brines could impact the overall selective adsorption of a target metal ion. In other words, metal ions of the same charge would compete for the available active sites present on the adsorbent and hinder the adsorption of some metals. Therefore, the selective adsorptive potential of the RDP, RDP-FC-Cu, and RDP-FC-Ni was examined. This was achieved by applying the adsorption studies on the collected reverse osmosis brine sample at the optimum pH of 6. Also, similar experimental conditions to previous adsorption experiments were applied. The adsorbent dosage of either RDP, RDP-FC-Cu, or RDP-FC-Ni was kept at 0.05 g. The solution volume of RO brine was 50 mL and the reaction temperature was kept at 35 ℃. The results of the study showed that all adsorbents achieved unique adsorptive capabilities towards the various metals in the brine sample. For example, the highest quantity of potassium and sodium was adsorbed by RDP. On the other hand, a full recovery of calcium ions was achieved by RDP-FC-Cu and RDP-FC-Ni while RDP adsorbed a quantity of 77,065 mg/L of the initial amount of 77,120 mg/L. Moreover, the RDP-FC-Cu and RDP-FC-Ni showed full recoveries for lithium while RDP achieved a recovery of 43.85 mg/L from an initial amount of 44.2 mg/L. Furthermore, the three adsorbents adsorbed the full quantities of cesium, zinc, lanthanide, barium, lead, strontium, and vanadium from the RO brine sample. The results showed that the adsorbents could be effectively utilized to adsorb a variety of metal ions from the RO brine. The significant result is that RDP-FC-Cu and RDP-FC-Ni are more efficient in fully recovering lithium ions from the RO brine. This provides scientists a sustainable and successful adsorption process of the target metal despite the presence of many competing ions in the sample.

## Conclusion

The desalination of seawater produces great amounts of concentrated brine. Lithium has been reported to be found in seawater reverse osmosis brine and it could be used in a variety of fields like lithium-ion batteries. The utilization of roasted date pits, as well as chemically modified form with potassium metal hexacyanoferrates for the recovery of lithium from a reverse osmosis brine in Qatar, was investigated. All the prepared materials (RDP, RDP-FC-Cu, and RDP-FC-Ni) showed great stability, porosity, functional groups, chemical structure, and composition as well as other adsorptive characteristics. Unique physical and chemical changes occurred on the RDP-FC-Cu and RDP-FC-Ni such as higher surface area, functional groups as well as composition and structure. Moreover, two of the most influential factors that affect the adsorption processes were studied namely, the effect of solution pH, as well as metal-ion concentration. The adsorption of lithium onto the three adsorbents favored a pH of 6 as well as increasing metal concentration. However, it was found that RDP-FC-Cu and RDP-FC-Ni achieved greater adsorption capacities and removals for lithium than RDP. Interestingly, RDP-FC-Cu accounted for slightly higher adsorption for lithium due to many factors like higher pore radius and volumes, the cubic lattice structure, similar hydrated ionic radius of lithium, copper, and potassium, which facilitated the metal substitution and complexation onto RDP-FC-Cu. Moreover, the significantly high desorption capabilities of the adsorbents provide scientists with opportunities for their multiple uses. The composites achieved full recoveries of lithium from the RO while RDP achieved significantly high adsorption. This stresses the main aim of this study, which is to create sustainable opportunities for the safe management of desalination brine and agricultural wastes.
